# YouTube as a Source of Information on Pilonidal Sinus Disease: A Reliability and Quality Analysis

**DOI:** 10.7759/cureus.34792

**Published:** 2023-02-09

**Authors:** Kayhan Ozdemir, Ali Muhtaroğlu

**Affiliations:** 1 General Surgery, Urgup State Hospital, Nevsehir, TUR; 2 General Surgery, Giresun University Faculty of Medicine, Giresun, TUR

**Keywords:** quality, reliability, information, video, youtube, internet, pilonidal sinus

## Abstract

Aim

This study aimed to assess the quality and reliability of the information in YouTube videos regarding pilonidal sinus disease (PSD).

Methods

A total of 100 most-viewed videos on PSD were included in the analysis by two general surgeons. Video duration and content, date of upload, qualification of the video uploaders, and number of daily and total view, like, and comment counts were analyzed. We grouped the videos as those uploaded by healthcare professionals and non-professionals. The quality of the video contents was assessed with the Global Quality Scale (GQS) and the reliability of the video content with the DISCERN scoring system.

Results

Eighty-five (85%) videos were uploaded by professionals and 15 (15%) videos by laypersons. The average DISCERN score of the reviewed video contents was 3.18 ± 1.23, and the average GQS score was 3.39 ± 1.24. In the overall subjective evaluation, the mean DISCERN value was found as 4.01 ± 1.24 and the mean GQS value as 4.25 ± 1.25 in the useful videos. The mean DISCERN value was found as 2.32 ± 1.22 and the mean GQS value as 2.48 ± 1.25 in the misleading videos.

Conclusion

Our results showed that most of the YouTube videos on PSD were loaded by healthcare professionals. However, the mean quality and reliability scores were lower in videos uploaded by laypersons. Given the prevalence of PSD, physicians should be promoted for uploading accurate and professional video contents to direct patients to the right solutions for their problems.

## Introduction

Pilonidal sinus disease (PSD) is an acquired, benign disease of the natal cleft. PSD usually affects young males aged 15-30 years with a reported incidence of 6/100,000 and a prevalence of 8.3% [1]. PSD usually manifests as a chronically discharging abscess or sinus tract with a single or multiple pits. Its etiology is multifactorial including male sex, obesity, family history, dietary habits, jobs requiring prolonged sittings, poor hygiene, and excessive hair in the natal cleft region [2].

Pain, local sepsis, and discharge caused by PSD have a significant negative impact on patients’ quality of life, although it can be easily treated with minimally invasive surgery. Many patients with PSD hesitate to seek medical help for the disease because it is located in a private region of the body. Therefore, patients with PSD may often search for a remedy before seeing a doctor. In fact, increasingly more people seek a solution for their health-related problems on the Internet rather than presenting to a healthcare center.

Today, 80% of Internet users search for medical information using online platforms on the Internet [3]. Particularly, patients with specific diseases trust Internet-based sources, and 75% report that their decision for treatment is based on information they have obtained on the Internet [4]. YouTube (Alphabet Inc., Mountain View, CA, USA), a popular video-sharing platform, is the second most commonly visited website globally following Google [5]. YouTube has currently two billion users with 42.9% of all global Internet users accessing YouTube monthly. More than a billion hours of content is viewed on YouTube every single day (YouTube statistics; available at https://backlinko.com/youtube-users).

Given that digital health literacy is increasing day by day, YouTube can provide an opportunity to offer the public health-related information and direct people with health problems to accurate medical sources. A recent study showed that hashtags of some specific diseases on Twitter, another social media platform, often referenced YouTube, highlighting that online traffic is largely driven to YouTube [6]. However, the information shown on YouTube lacks scientific rigor because anyone can upload such content free of charge and audit [7]. Indeed, YouTube hosts a large volume of health-related content of uncertain value from diverse sources [8]. Inappropriate video contents are often uploaded because YouTube does not have a policy to question the credibility and qualification of video uploaders [9]. Studies increasingly draw attention to misleading, low-quality, health-related content uploaded to YouTube by non-medical sources [10-13]. In a systematic review of 18 studies, it was found that YouTube includes misleading and conflicting health-related video content, as well as high-quality information [14]. This study aimed to assess the quality and reliability of information in YouTube videos regarding PSD.

## Materials and methods

Search strategy on YouTube

This study was conducted by two experienced general surgeons as a reliability and quality analysis of information on YouTube videos concerning pilonidal sinus disease at 07-09/10/2022. The search terms were decided by the consensus of the researchers as “pilonidal sinus,” “pilonidal sinus disease,” “pilonidal cysts,” and “ingrown hair” based on the most common trends on Google search engine. From filtering options of the YouTube platform, the video contents were sorted by relevance option. It has been reported in previous studies that 90% of YouTube users view the first results [15,16]. Accordingly, the most-viewed 100 videos were analyzed. The links of these videos were entered into the Microsoft Excel program (Microsoft® Corp., Redmond, WA, USA). Among the filtered outcomes, advertisements, duplicate videos, non-English videos, and those lasting longer than one hour were not included in the analysis. The analysis of the reviewed videos was performed by the two researchers in separate settings but at the same time in order to avoid possible bias.

Data retrieval

The data used in this study included video content, video duration, qualification of the uploaders, time of upload, the number of daily views, and the total view, like, and comment counts. Since YouTube removed the number of dislikes, this was not included in the analysis. We further grouped the videos as the videos uploaded by healthcare professionals and those uploaded by non-professionals. Accordingly, the videos that had been directly uploaded by hospital channels and health channels were considered uploaded by professionals because the narrators of these videos were physicians. The other uploaders were considered non-healthcare professionals. The contents of the videos reviewed included general information about PSD, surgical technique, alternative medicine, patient experience, narration, and differential diagnosis. Narrative videos included a narrator (mostly doctors) without displaying any procedure or other images.

Evaluation of the videos

After separate scoring of the videos, subjective assessment was made by the consensus of the two researchers, and all videos were classified as useful and misleading, independently from the scores given. The quality of the video contents was assessed with the Global Quality Scale (GQS) and the reliability of the video content with the DISCERN scoring system. These two online content assessment scales have been commonly utilized in numerous studies investigating YouTube videos on different diseases [10-13]. The videos were assessed independently by two general surgeons.

DISCERN scale

DISCERN is a scoring scale for evaluating the reliability of consumers’ health-related information on treatment options. In the present study, we used the shortened version of the DISCERN tool adapted by Singh et al. from the original form [17]. The modified DISCERN include five items, each scored using a five-point Likert scale. The DISCERN items question the reliability of information sources, aims, bias, areas of uncertainty, and additional sources. A DISCERN score of >3 indicated good, a score of 3 showed moderate, and a score of <3 pointed out to a weak video content [11].

GQS

GQS is a tool developed by Bernard et al. in order to evaluate the quality of a video content based on the utility of the healthcare information offered in the video [18]. GQS consists of five items that evaluate the quality of information with a five-point Likert scale. The quality of a healthcare content is evaluated with scores between 1 (very poor) and 5 (excellent).

Statistical analysis

The results obtained in this study were analyzed using Statistical Package for Social Sciences (SPSS) version 24.0 (IBM SPSS Statistics, Armonk, NY, USA) software package. The distribution of the data was evaluated using the Kolmogorov-Smirnov test. Since the variables were non-normally distributed, the Mann-Whitney U test was used in the comparison of the continuous variables, and the chi-square test was used in the comparison of the categorical variables between the groups. The Kruskal-Wallis test was used in the comparison of the DISCERN and GQS scores based on the video sources. Continuous variables were given as mean ± standard deviation, while categorical variables were expressed as frequency and percentage (n and %). The calculation of the agreement between the two observers was made using Cronbach’s alpha coefficients. A p < 0.05 was considered statistically significant.

## Results

**Figure 1:**
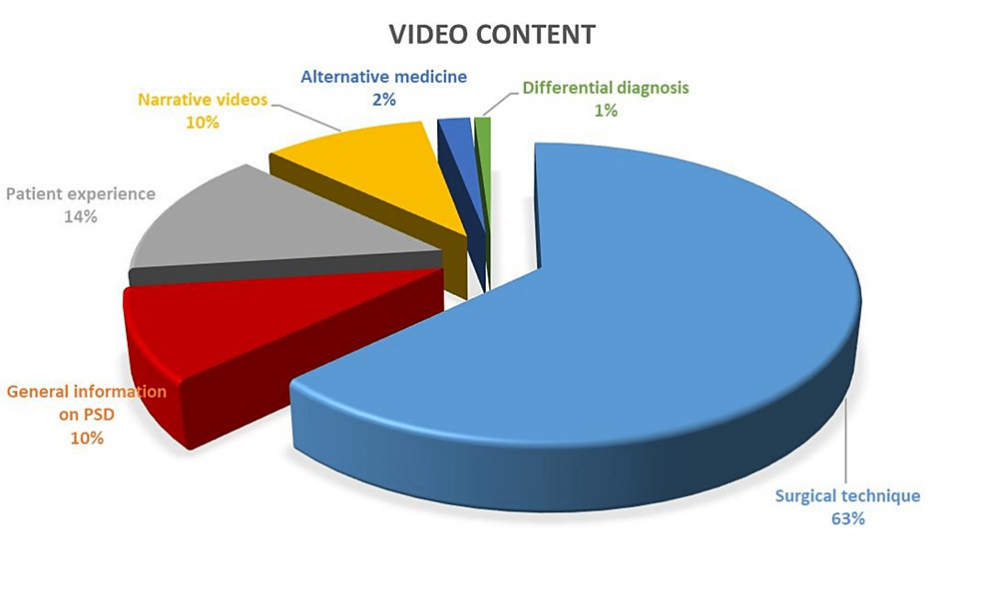
Distribution of the reviewed YouTube videos by content PSD: pilonidal sinus disease

In this study, we evaluated a total of 100 YouTube videos on PSD in terms of the reliability and quality of the information they offer. The total view count of these 100 videos was approximately 40 million with about a 10-hour duration. Video content analysis revealed that 63 (63%) videos included surgical technique, 14 (14%) videos included patient experience, 10 (10%) videos included general information about PSD, 10 (10%) videos included only narration without displaying images, two (2%) videos included alternative medicine for the treatment of PSD, and one (1%) video contained differential diagnosis (Figure 1).

The uploaders were found as medical doctors in 57 (57%) videos, while patients and laypersons uploaded 15 (15%) videos, hospital channels uploaded 14 (14%) videos, and health channels uploaded 14 (14%) videos. We further divided all videos in two groups as the videos uploaded by healthcare professionals and laypersons. Accordingly, 85 (85%) videos were uploaded by professionals and 15 (15%) videos by laypersons.

The average length of the videos was found as 5.63 ± 4.86 minutes. The mean video duration was measured as 5.64 ± 4.86 minutes in the videos uploaded by the professionals and 5.59 ± 4.31 minutes in those uploaded by laypersons. There was no statistically significant difference in video duration between the two groups (p > 0.05). The oldest video was uploaded on 30/12/2007 and the last newest video on 30/10/2021. The mean duration since the upload was found as 1,554.23 ± 1,080.74 days. The average daily views and comment and like counts of the videos are presented in Table 1.

**Table 1:** Basic characteristics of the videos SD: standard deviation

Feature	Daily Views	Comments	Likes
	Mean ± SD	Mean ± SD	Mean ± SD
Image Type			
Real (n = 93)	238 ± 504	266 ± 460	1,013 ± 1,981
Animation (n = 7)	155 ± 514	83 ± 468	768 ± 2,017
Uploaders			
Medical Doctors (n = 57)	250 ± 504	223 ± 460	846 ± 1,981
Hospital Channels (n = 14)	304 ± 532	277 ± 483	1,610 ± 2,087
Health Channels (n = 14)	197 ± 509	61 ± 464	850 ± 2,000
Patients (n = 15)			
Professionals Versus Laypersons			
Professionals (n = 85)	250 ± 504	205 ± 460	972 ± 1,981
Non-professionals (n = 15)	131 ± 474	422 ± 481	1,126 ± 1,707
Video Content			
Surgical Technique (n = 63)	318 ± 507	225 ± 462	1,120 ± 1,990
Patient Experience (n = 14)	102 ± 474	520 ± 481	1,256 ± 1,707
General information (n = 10)	44 ± 507	80 ± 462	220 ± 1,990
Narrative (n = 10)	84 ± 453	114 ± 461	596 ± 2,033
Alternative Medicine (n = 2)	184 ± 58	52 ± 603	873 ± 1,179
Differential Diagnosis (n = 1)	125	325	1,500

The 100 videos enrolled in this study were subjected to an overall evaluation following the scoring process by the two researchers. In the case of disagreement, the view of a third general surgeon was requested, and the decision was made on the consensus of the three surgeons. Accordingly, 49 (49%) videos were evaluated as misleading and 51 (51%) as useful regardless of the uploaders.

The quality of the video contents was assessed with the Global Quality Scale and the reliability of the video content with the DISCERN scoring system. The average DISCERN score of the videos was found as 3.18 ± 1.23, and the average GQS score as 3.39 ± 1.24. In the overall subjective evaluation, the mean DISCERN value was found as 4.01 ± 1.24 and the mean GQS value as 4.25 ± 1.25 in the useful videos. The mean DISCERN value was found as 2.32 ± 1.22 and the mean GQS value as 2.48 ± 1.25 in the misleading videos. Table 2 shows the DISCERN and GQS scores based on the uploaders and video contents.

**Table 2:** Distribution of the DISCERN and GQS scores GQS, Global Quality Scale; SD, standard deviation

Feature	n (%)	DISCERN	GQS	P value
		Mean ± SD	Mean ± SD	
Uploaders				
Professionals	85 (85)	3.47 ± 1.23	3.70 ± 1.24	<0.001
Laypersons	15 (15)	1.53 ± 1.34	1.60 ± 1.38	
Video Content				
Surgical Technique	63 (63)	3.52 ± 1.22	3.75 ± 1.25	<0.001
Patient Experience	14 (14)	1.57 ± 1.34	1.64 ± 1.38	
General Information	10 (10)	3.10 ± 1.24	3.25 ± 1.25	
Narrative	10 (10)	3.70 ± 1.14	3.95 ± 1.17	
Alternative Medicine	2 (2)	1.00	1.00	
Differential Diagnosis	1 (1)	4.50	5.00	

According to the DISCERN results, 41 (41%) videos were of poor quality, nine (9%) were of medium quality, and 50 (50%) were of good quality. Based on the GQS results, eight (8%) videos were of very poor quality, and 20 (20%) videos were excellent. Cronbach’s alpha coefficient was used to evaluate the agreement between the two raters. As a result, there was an excellent agreement between the two surgeons in terms of the GQS and DISCERN scores (Table 3).

**Table 3:** DISCERN and GQS scores given by the researchers separately GQS, Global Quality Scale; SD, standard deviation

	Mean ± SD	p	r	Cronbach’s alpha
DISCERN 1	3.14 ± 1.22	<0.01	0.895	0.902
DISCERN 2	3.12 ± 1.24			
GQS 1	3.42 ± 1.32	<0.01	0.903	0.925
GQS 2	3.46 ± 1.34			

**Figure 2:**
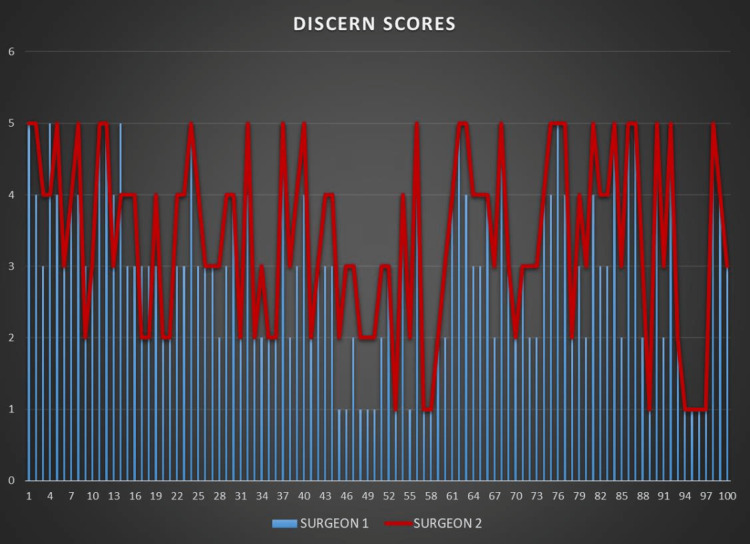
The DISCERN scores given by the two researchers

**Figure 3:**
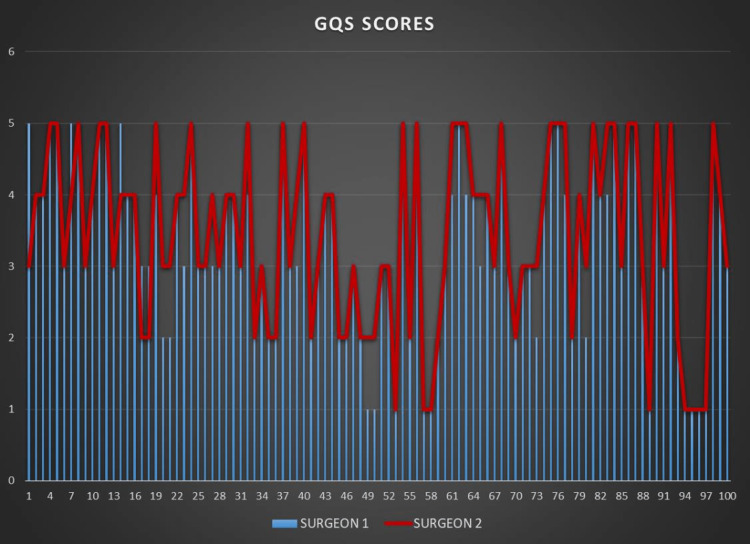
The GQS scores given by the two researchers GQS: Global Quality Scale

The DISCERN and GQS scores given by the two researchers are shown in Figures 2-3.

## Discussion

Today, patients and their relatives are increasingly using the Internet (Google, YouTube, WebMD, Mayo, etc.) as a source of information to seek help for their health problems, to share experiences, and even to buy treatment [19]. In a survey, it was found that the Internet is the third most trustworthy source of health-related information following physicians and official health institutions [3]. As one of the most preferred video-sharing platforms, YouTube hosts a lot of videos about general information, diagnosis, treatment, alternative options, and prevention of various diseases [14]. The first YouTube analysis study was conducted by Keelan et al. to perform research on YouTube videos regarding immunization [20]. Currently, there is a surge of health-related YouTube videos as evidenced in PubMed. A search we made on PubMed on 09/10/2022 using the search term “YouTube” yielded 2,088 results. While YouTube provides free video content to website users, it lacks a control mechanism for the quality and accuracy of the videos. For example, in a study investigating YouTube videos on smoking cessation, the uploaders were found to be everyday YouTube users, as well as healthcare professionals, pointing out the seriousness of the issue. Patients with specific diseases including PSD refer to YouTube with hesitation to see a doctor for their problem due to the fear of the violation of their privacy.

In this study, we assessed the reliability and quality of the 100 most-viewed YouTube video contents regarding pilonidal sinus disease. The 100 most-viewed video contents were evaluated by two experienced general surgeons independently. The total view count of the videos was about 40 million with approximately 10-hour duration, suggesting a public interest on the issue. The most-viewed video was uploaded by an Indian doctor. The video (four minutes and 56 seconds) was viewed 10,569,264 times with 11,000 likes.

In this study, the video contents were uploaded by doctors, hospital channels, health channels, patients, and laypersons. However, because the narrators of the videos were again doctors, in those uploaded by hospital and health channels, we divided the uploaders into two wider groups as professionals and laypersons. Accordingly, in our study, 85% of the video contents were uploaded by healthcare professionals and 15% by non-professionals. In a study from Spain investigating YouTube as a source of influenza vaccine information, Hernández-García and Giménez-Júlvez reported that only 23% of the video contents were uploaded by health professionals [5]. In another study investigating YouTube videos on male infertility, 50% of the videos were uploaded by healthcare professionals [6]. Different results among the studies might have resulted from different characteristics between diseases. It is obvious that the results of specific diseases are not expected to be similar to the common diseases. On the other hand, the higher rates of videos uploaded by professionals in our study reflect the fact that only little information or misinformation is known about PSD by the public.

In our study, the majority (63%) of videos contained information about surgical technique. We think the reason for this is that surgery is the only definitive solution in PSD. Other studies have reported different rates about the video content. In a study evaluating YouTube videos pertaining to rotator cuff tears, Kuru et al. reported that 14% of video contents were about surgical methods. Again, these rates differ among the studies according to the topic of search [21].

We intend to analyze our DISCERN and GQS scores between professionals and laypersons and to compare our results with the most recent studies in the literature. In the present study, the mean DISCERN score was 3.47 ± 1.23 for the videos uploaded by healthcare professionals and 1.53 ± 1.34 for the videos uploaded by laypersons. The mean GQS score was calculated as 3.70 ± 1.24 for the videos uploaded by healthcare professionals and 1.60 ± 1.38 for the videos uploaded by laypersons. When we analyzed the high difference between the professional and layperson videos, we found out some striking points. In almost all videos uploaded by patients or laypersons, alternative but not scientific methods were mentioned for the treatment of PSD. Even in some videos, there were only images with music in the background without any explanation. People in these videos were discouraging PSD patients from seeking medical help for their problem.

Similar results have been reported in the most recent studies. In a study of YouTube videos on contact dermatitis, the mean GQS score was reported as 4.5% in the video contents uploaded by medical sources and 2% in those uploaded by non-medical sources [8]. Chang and Park investigated YouTube videos on dysphagia and reported significant differences between the low- and high-quality videos in terms of DISCERN scores (3.39 ± 0.74 versus 1.6 ± 1.14) [14]. The results of Chang and Park’s study are close to our findings. In the study of YouTube videos on surgical treatment of uterine leiomyomas, Ergul found the mean DISCERN score as 2.7 ± 0.9 for the informative video contents and 1.6 ± 0.9 for patient experience. In the same study, the mean GQS scores were found as 3.7 ± 1.0 and 2.4 ± 1.1, respectively [22]. Our results are in general consistent with the results of other studies in terms of the reliability and quality of YouTube video contents.

Study limitations

First, the focus was only on English-language videos. Video contents in other languages might change the results. Second, the video evaluation was a subjective process, but the inter-observer agreement was excellent. Finally, further studies could include more observers of varying backgrounds such as patients and different age groups in order to minimize subjective bias in the video scoring process.

## Conclusions

Our results showed that most of the YouTube videos on PSD were uploaded by healthcare professionals. However, the average quality and reliability scores were low even for some video contents uploaded by physicians. Given the prevalence of PSD worldwide, doctors should be promoted to upload accurate and more professional video contents to direct patients to the right solutions for their problems. Finally, YouTube should improve its privacy and community guidelines to include supervision of healthcare-related videos before they can be published.
